# The late positive potential indexes a role for emotion during learning of trust from eye-gaze cues

**DOI:** 10.1080/17470919.2015.1017114

**Published:** 2015-11-16

**Authors:** Luis R. Manssuer, Mark V. Roberts, Steven P. Tipper

**Affiliations:** ^a^ School of Psychology, Bangor University, Gwynedd, UK; ^b^ Department of Psychology, University of York, York, UK

**Keywords:** EEG, Emotion, Faces, Gaze, Trustworthiness, N250

## Abstract

Gaze direction perception triggers rapid visuospatial orienting to the location observed by others. When this is congruent with the location of a target, reaction times are faster than when incongruent. Functional magnetic resonance imaging studies suggest that the non-joint attention induced by incongruent cues are experienced as more emotionally negative and this could relate to less favorable trust judgments of the faces when gaze-cues are contingent with identity. Here, we provide further support for these findings using time-resolved event-related potentials. In addition to replicating the effects of identity-contingent gaze-cues on reaction times and trust judgments, we discovered that the emotion-related late positive potential increased across blocks to incongruent compared to congruent faces before, during and after the gaze-cue, suggesting both learning and retrieval of emotion states associated with the face. We also discovered that the face-recognition-related N250 component appeared to localize to sources in anterior temporal areas. Our findings provide unique electrophysiological evidence for the role of emotion in learning trust from gaze-cues, suggesting that the retrieval of face evaluations during interaction may take around 1000 ms and that the N250 originates from anterior temporal face patches.

High visual acuity costs the brain space and energy. Consequently, the restricted visual field size of the fovea has to be constantly moved via attention and oculomotor systems for a detailed representation of objects in different spatial locations to be maintained. Such refixations are readily perceivable by others aided by the high contrast between the human iris and sclera (Kobayashi & Kohshima, 1997, 2001) providing valuable visual cues to objects of emotional significance and likely targets for action. As such, eye-gaze direction detection features prominently in models of social cognition and appears to be the most important cue to “social attention” (Baron-Cohen, 1995; Emery, 2000; Langton, Watt, & Bruce, 2000). Indeed, experimental evidence shows humans are adept at fine perceptual discrimination of gaze direction (Anderson, Risko, & Kingstone, 2011; Gibson & Pick, 1963) and at shifting attention to detect objects in the line of others’ sight (Langton, O’Donnell, Riby, & Ballantyne, 2006), two functions which neuroimaging has associated with the anterior superior temporal sulcus (Carlin, Calder, Kriegeskorte, Nili, & Rowe, 2011) and intraparietal sulcus (Ramsey, Cross & Hamilton, 2011), which have been proposed to form part of a network interfacing gaze perception with attentional orienting (Carlin & Calder, 2013).

The gaze processing system is clearly illustrated in studies of gaze-cueing, which show reaction times to targets presented laterally to a face are quicker when the face gazes toward the target, congruently, compared to when gazing away, incongruently (Driver et al., 1999; Friesen & Kingstone, 1998; Frischen, Bayliss, & Tipper, 2007). Gaze-cueing effects are robust and reflexive. They are not affected by the knowledge that the target is more likely to appear in the opposite direction of the gaze (Driver et al., 1999), not reduced by visual and verbal working memory load (Law, Langton, & Logie, 2010), can occur under high perceptual load conditions, such as in rapid serial visual presentation, and without awareness, under flash suppression and backward masking (Sato, Okada, & Toichi, 2007; Xu, Zhang, & Geng, 2011). Given the reflexivity of gaze-cueing, it is not surprising that such cues can be used to mislead others into attending away from important stimuli (Klein, Shepherd, & Platt, 2009). These deceptive gaze-cues also influence trust judgments of the gazer. When particular faces consistently gaze away from target objects, they are judged less trustworthy than faces that consistently gaze toward, an effect that is larger for happy compared to angry and neutral faces (Bayliss, Griffiths, & Tipper, 2009; Bayliss & Tipper, 2006). However, it is unclear as to how gaze-cues translate into changes in trust judgments.

The gaze-cueing effects on trust could be mediated by emotion. Indeed, Dunn and Schweitzer (2005) found that when participants were induced to feel angry, they trusted others less than when they were induced to feel happy. Evidence also suggests that gaze-cues can elicit emotions. Using fMRI, two studies have employed a paradigm in which participants were instructed to either follow or not follow the gaze of another individual toward an object, or were instructed to direct the other individual’s gaze toward an object using their own gaze, which was either reciprocated or not reciprocated by the other individual (Gordon, Eilbott, Feldman, Pelphrey & vander Wyk, 2013; Schilbach et al., 2010). These studies found that self-initiated joint attention compared to non-joint attention was rated more pleasurable, less difficult and elicited greater activity in the amygdala and striatum, neural structures associated with reward. The latter region correlated with subjective pleasantness ratings. Thus, incongruent gaze-cues may be experienced as less pleasant than congruent cues. However, while fMRI may be useful at localizing subcortical activity, it has poor temporal resolution and can suffer from signal dropout in orbitofrontal regions adjacent to air-filled chambers in the skull, regions that are heavily implicated in emotion (Rolls & Grabenhorst, 2008) and social valuation (Campbell-Meiklejohn et al., 2012).

In contrast, event-related potential (ERP) analysis of electro-encephalographic (EEG) data provides both high temporal resolution and sensitivity to affective processes. In particular, the late positive potential component (LPP or LPC), a slow wave beginning around 300–400 ms on frontal, central and occipitoparietal sensors, and which usually remains sustained for the duration of the stimulus, has been shown to be sensitive to stimuli with positive and negative valence compared to neutral stimuli (Cuthbert, Schupp, Bradley, Birbaumer, & Lang, 2000; Schupp et al., 2000). In combined EEG–fMRI studies, the LPP has been shown to relate to concurrent activity in brain regions involved in visual/attentional processing such as lateral occipital, parietal and inferotemporal cortices and emotion regions such as the orbitofrontal cortex, insula, anterior cingulate cortex, ventral striatum and amygdala (Liu, Huang, McGinnis-Deweese, Keil, & Ding, 2012; Moratti, Saugar, & Strange, 2011; Sabatinell, Lang, Keil, & Bradley, 2007; Sabatinelli, Keil, Frank, & Lang, 2013).

The LPP is believed to reflect processing of, attention to, and memorization of, the emotional content of stimuli (Hajcak, Mcnnamara & Olvet, 2010). As the LPP is larger for images containing faces compared to objects and scenes not containing faces, this suggests that faces hold a significance that is unparalleled by other classes of stimuli (Ferri, Weinberg, & Hajcak, 2012; Weinberg & Hajcak, 2010). This appears to be due to the multiple, salient, affectively valent social cues that characterize faces. Experiments using more controlled face stimuli have shown the LPP to be modulated by attractiveness (Wiese, Altmann, & Schweinberger, 2014), trustworthiness (Marzi, Righi, Ottonello, Cincotta, & Viggiano, 2014; Yang, Qi, Ding, & Song, 2011) and expressions (Smith, Weinberg, Moran, & Hajcak, 2013).

In this study, we examined the role of emotion in learning trustworthiness from gaze-cueing by recording high-density EEG during an identity-contingent gaze-cueing task. Given its role in emotion processing, we hypothesized that the LPP would index the learning of trust judgments from gaze-cues. Unlike evoked sensory components, such as the N170, the LPP is much more variable in the time domain and differences between conditions can occur in brief time-windows from 300 ms after stimulus-onset until stimulus-offset (Hajcak et al., 2010). Thus, traditional ERP analyses may risk overlooking important effects. This is especially the case since our paradigm is relatively novel to EEG and because of the long duration and multiple trial periods in our design. Therefore, we used the statistical parametric mapping (SPM) approach (Kilner & Friston, 2010), in which analysis is performed on interpolated 3D images of scalp activity over time and corrected for multiple comparisons with random-field theory (RFT) to identify clusters of significant activity localized in time on the scalp. This approach avoids the bias associated with traditional ERP analyses (Ibanez et al., 2012; Kilner, 2013; Kriegeskorte, Simmons, Bellgowan, & Baker, 2009), preserves the high resolution and dimensionality of the data while allowing us to detect significant effects with no a priori prediction about where and when these effects would occur.

In addition to analyzing effects within the gaze-cueing paradigm, we also examined neural responses to images of the faces before and after gaze-cueing trials to examine whether effects of learning trust from gaze-cues modulates face related ERP components such as the P1, N170 and N250, which have been implicated in the perceptual processing, structural coding and recognition of faces, respectively (Schweinberger, 2011). The N250, a negative deflection at 250 ms on occipitotemporal electrodes, which is greater when preceded by an image of the same face (Schweinberger, Huddy, & Burton, 2004; Schweinberger, Pickering, Jentzsch, Burton, Kaufmann, 2002) or when the face has become familiar by repeated viewing (Pierce et al., 2011; Tanaka, Curran, Porterfield, & Collins, 2006; Zimmerman & Eimer, 2013), was of particular interest given its proposed role in face recognition and may link particular faces with particular traits. Thus, recording neural activity before gaze-cueing provides an initial baseline to compare with after cueing, when faces are familiar and trust is learned. We also followed up strong effects on the scalp with Bayesian 3D source reconstruction using multiple sparse priors (MSPs) (Friston et al., 2008), which previous studies have used to estimate the source of value-related signals (Harris, Adolphs, Camerer, & Rangel, 2011) and face-responsiveness (Henson, Mouchlianitis, & Friston, 2009).

## METHOD

### Participants

There were 26 participants overall of which 24 were female and all right handed. Participants were volunteers from Bangor University with an average age of 22 (*SD =* 4). All were neurologically normal with normal or corrected-to-normal vision and received course credit or £15 for taking part. The university ethics board approved all procedures.

### Stimuli and apparatus

The 16 faces used in the experiment were eight females and eight males adapted from the NimStim face database (Tottenham et al., 2009). A previous study has shown the effect of gaze-cues on learning trust is greatest when the faces express happiness as opposed to a neutral expression (Bayliss et al., 2009). In addition, mildly happy faces have typically been used as neutral baseline comparison stimuli in experiments of facial expression perception due to the tendency for people to smile slightly in normal social interactions, whereas faces that show no contraction of the facial muscles could appear hostile (Mattavelli et al., 2014). Therefore, all faces were made to appear to have a mildly happy facial expression. The mildly happy faces were created by morphing a neutral version of each face with a happy version to create 20 frames varying from neutral to happy. A set of 10 observers were then asked to adjust each face to the point at which it could just be detected as happy. The average frame chosen was used in the experiment. It is noteworthy that a previous experiment using the same faces as the current experiment found that happy expressions did not elicit a larger LPP compared to neutral (Smith et al., 2013). Thus, it is unlikely that the happy expression of the face will produce a ceiling effect in the LPP that limits any responses related to gaze.

The faces were divided into two groups, A and B. The faces in each group were matched for visual appearance and ratings of trust and attractiveness in a previous study (Bayliss et al., 2009). Leftward and rightward gaze-cues were created by moving the irises into the left and right hand corners of the eyes.^1^ ^1^In addition, during cueing, the pupil size of the faces was manipulated to be larger for congruent faces and smaller for incongruent faces. Pupil size is related to emotional arousal and interest in visual stimuli (Bradley, Miccoli, Escrig, & Lang, 2008; O’Doherty, Buchanan, Seymour, & Dolan, 2006). In the current experiment, pupil size was manipulated as an attempt to enhance effects of validity on trust learning under the assumption that small pupils signal a lack of interest and larger pupils greater interest and these states may be recognized and integrated with gaze-cueing information. However, when comparing our data to an identical unpublished study where pupil was not manipulated we found no difference with the current experiment in gaze-cueing reaction time effects or on learning of trust based on gaze-target contingencies. Therefore we will not discuss pupil size manipulation further. The faces subtended 12.2° × 12.5° in the cueing phase and 15.2° × 15.6° in the viewing and rating phases. The target stimuli were a set of 32 garage and 32 kitchen objects. There were 16 unique objects in each category, which were in two different orientations. All were blue colored and presented centrally to the left- or right-hand side of the face in line with the eyes subtending 7.1° × 5.7°. The experiment took place in a Faraday cage to shield external electromagnetic noise and it was maintained at a slightly cool temperature to avoid sweat waves. Participants sat in a comfortable chair at a distance of approximately 100 cm from the screen. The experiment was run using E-Prime 1.0 (Psychology Software Tools Inc., Sharpsburg, PA, USA) on a 24″ Samsung SyncMaster BX2431 LED display, which was 342 × 569 mm in dimensions and had a 500 Hz refresh rate.

### EEG recording

Electroencephalographic data were collected continuously using a 128 channel BIOSEMI Active Two system at 2048 Hz. All participants washed their hair with baby shampoo before suitable sized electrode caps were fitted. Gel was injected into each of the receptor sites before attaching Ag–AgCl active electrodes. Horizontal eye movements were recorded with electro-ocular (EOG) electrodes placed on the outer canthi of both eyes and vertical eye movements were recorded with two electrodes each placed infraorbitally and supraorbitally around the left eye. The EEG was monopolar referenced online using a common mode rejection active electrode.

## DESIGN AND PROCEDURE

### Gaze-cueing phase

**Figure 1.**
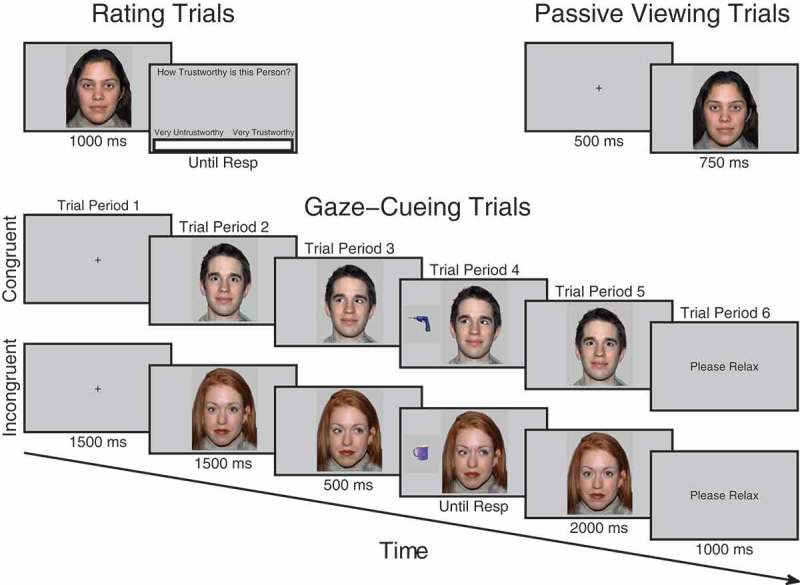
Trial procedure for trust rating, passive viewing and cueing trials. On trust rating trials before and after viewing phases, participants observed each face for 1000 ms after which a visual analog scale (VAS) appeared requiring them to click the point on the scale which represented how trustworthy they judged the face to be. On passive viewing trials, which occurred immediately before and after the cueing phase, participants viewed a fixation cross for 500 ms followed by a face for 750 ms. During cueing trials, participants saw a fixation cross for 1500 ms, followed by a face looking directly for 1500 ms after which it shifted its gaze direction to the left or right for 500 ms, at that point a kitchen or garage target object was presented. When participants classified the object with a key-press it disappeared and the gaze returned to look toward the participants for another 2000 ms.

For half of the participants, faces in group A were designated congruent and would consistently look toward targets whereas faces in group B were incongruent and would consistently look away from targets. For the other half of participants, the contingencies were reversed. Participants initiated each trial with the space bar, a fixation cross appeared for 1500 ms followed by a directly gazing face for 1500 ms. The face then changed gaze direction and remained for 500 ms after which an object appeared to the left- or right-hand side of the face and disappeared as soon as a response was made or until 3000 ms elapsed. When a response was made, the object disappeared and the face gazed directly again for 2000 ms (see Figure 1). At the end of the trial, participants saw a screen saying *Please Relax* for 1000 ms. A 500 ms stimulus onset asynchrony (SOA) between gaze-cue and target object was used to ensure that it was long enough for gaze to be most strongly encoded and produce measurable ERPs but at the same time short enough to reflexively cue attention (Friesen & Kingstone, 1998). This SOA has also been used in all other studies of identity-contingent gaze-cueing and trust (Bayliss et al., 2009; Bayliss & Tipper, 2006; Rogers et al., 2014), facilitating comparison. We did not vary SOA, as this would not allow for a sufficient number of ERP trials to be calculated in the trial period when the gaze-shift occurred. A previous unpublished experiment using exactly the same design and SOA as the current study showed that the gaze-cueing effect was not reduced after five blocks of learning. Thus, at this SOA, participants do not use face identity and the gaze-shift to anticipate where the target will appear. There were five blocks in total each comprising 32 trials. Within each block, each face was presented twice, once gazing rightward and once gazing leftward. The order of trials within each block was randomized. Objects in each category were randomly sampled without repetition apart from when in the opposite orientation. Participants were told that their task was to classify the object not only as quickly as possible but also as accurately as possible and that the face was irrelevant to their task. Response keys were counterbalanced. Half of the participants pressed space bar for kitchen objects and the “H” button for garage objects whereas the other half did vice versa. Responses were made with the index finger on the “H” button and thumb on the space bar. If there was no response made within 3000 ms or if the response was incorrect, an error tone sounded for 1000 ms. Participants completed eight practice trials beforehand with unfamiliar faces that were not used in the main experiment.

### Passive viewing phases

Immediately before and after the cueing phase participants completed the passive viewing phases. In these phases, participants pressed space to initiate each trial. A fixation cross was presented for 500 ms followed by a face for 750 ms. After the face disappeared, participants were presented with a *Please Relax* screen for 1000 ms. There were 192 trials in total. Each face was intended to be repeated six times in each phase. However, randomization was repeated after every six faces had been presented as opposed to 16. This only introduced slight variability into the number of times each face was presented and did not differ significantly between conditions. See Appendix for further details.

### Trust rating phases

Before the initial viewing phase and after the end viewing phase, participants completed the rating phases. As in the viewing phases, both initial and final rating phases were the same. Each trial began when participants pressed space bar, at that point a fixation cross appeared for 1000 ms followed by a directly gazing face for 1000 ms and then a screen containing a VAS asking *How trustworthy is this person?* At this point, a cursor was visible on the screen and participants used the mouse to click along the scale at the point that represented how trustworthy they judged that person to be. The extreme left of the scale was labeled *Very Untrustworthy* and the extreme right of the scale was labeled *Very Trustworthy*. The center of the screen, therefore, represented neutral. When participants clicked on the scale, the computer recorded a rating between −100 and +100. The order of face identity on each trial was randomized.

The protocol of the experiment was modified to incorporate the attachment of the EEG cap and electrodes. After giving informed consent, participants were presented with all the kitchen and garage objects and asked to classify them in order to verify that they could do the gaze-cueing task properly. Feedback was given for incorrect responses. Participants then completed the initial rating phase, washed their scalp with baby shampoo, after that the EEG cap was fitted, electrode sites were filled with gel and electrodes were attached. Participants then completed a brief eye-movement task in which they were asked to make 20 leftward, rightward, upward and downward eye movements and eye blinks. This provided a clean template of ocular artifacts for later removal from the experimental data using independent components analysis (ICA). Participants then undertook the initial passive viewing phase followed by the cueing phase, end passive viewing phase and end trust rating phase. Afterwards, the EEG cap was removed; participants washed their hair and were debriefed.

### EEG data preprocessing and analysis

Data preprocessing was undertaken using Brain Vision Analyzer 2. The data were down sampled to 1024 Hz, filtered between 0.1 and 30 Hz (48 dB slope) and rereferenced to the average before being submitted to Infomax ICA to identify and remove eye movements and blink artifacts. The ICA was trained on each individual’s eye movement and blink activity recorded specifically for this purpose. Errors and outliers were then removed. For the cueing phase, all trials on which incorrect target object classifications occurred or on which participants took longer than 1500 ms to respond were removed. Each trial was then visually inspected for artifacts such as excessive EMG activity (blind to conditions). Bad channels were recalculated by interpolating between neighboring electrodes. All trials and trial periods were baseline corrected using the final 100 ms of the fixation periods. After preprocessing, all data were exported into SPM12 (Wellcome Department of Imaging Neuroscience, Institute of Neurology, London, UK) for statistical analysis allowing for the testing of effects over all time points and scalp sites while correcting for the family wise error (FWE) rate with RFT. Conditions of interest were epoched, averaged and converted into interpolated 3D images at a size of 32 × 32 voxels at each time point for cueing trials and 64 × 64 voxels at each time point for viewing trials. Images were smoothed with a 9 × 9 mm × 30 ms full-width half maximum (FWHM) Gaussian kernel. The images were entered into general linear models using a flexible factorial design. *F-*contrasts were used to test for significant effects. All effects were corrected for violations of sphericity and were only considered significant if clusters passed a cluster size threshold of *p *< .0001 RFT FWE corrected.

Significant effects on the scalp were followed up with distributed Bayesian 3D source reconstruction modeling using MSPs (Friston et al., 2008). This involved coregistering each individual’s sensor array to a template MNI brain using standard coordinates. The source space was modeled using a “canonical” mesh of the cortical surface and the boundary element model was used to account for volume conduction by the surrounding tissues whereby the cerebrospinal fluid, skull and skin are accounted for by tessellated meshes of different conductivities. Reconstruction entails computing the forward model of the lead fields from each cortical mesh vertex to the sensors and then performing inversion using the experimental data. Group inversion was employed in order to optimize the spatial covariance in reconstructed activity across subjects (Litvak & Friston, 2008; Litvak et al., 2011). For significant effects on the scalp, contrast waves of interest were computed for each subject and entered into the inversion, after which individual 3D images were generated by averaging across time windows of interest and submitted to a one-sample *t*-test.

### Data screening protocol

For analysis of the cueing ERP data, all trials on which participants made an error or failed to respond (*M *= 4.01%, *SD *= 3.08 of trials) were removed along with artifact ridden trials (*M *= 15.8% of trials, *SD *= 8.35%) and trials with reaction times above 1500 ms (*M *= 6.8%, *SD *= 8.37% of trials). Paired sample *t*-tests showed no significant difference in the number of errors between congruent and incongruent conditions, *t*(25) = .202, *p *= .841, 95% CIs [−.44 .54]. For trial period 4, where the duration depends upon the reaction time, extra trials were removed that were below 500 ms in duration (*M *= .55%, *SD *= 1.1%). After removal of errors, outliers and artifacts, there was no significant difference in the number of trials between incongruent and congruent conditions in trial period 4, *t*(1, 26) = .649, *p *= .522, 95% CIs [−1.84 3.530], and all other trial periods, *t*(1, 25) = .846, *p *= .40, 95% CIs [−1.6 3.8]. There was also no significant difference between incongruent and congruent conditions in terms of the number of artifact trials (*M* = 10.62%, *SD* = 9.8%) removed from the viewing analyses, *t*(25) = .597, *p* = .556, 95% CIs [−.49 .89]. For all ERP analyses, we collapsed across the factor of face gender, as this was not of primary interest to our hypotheses and also to retain a sufficient number of trials in the analysis. Trials with errors or reaction times exceeding 1500 ms or two standard deviations above or below each participants mean (*M *= 4.6%, *SD *= 1.5) were removed from the reaction time analyses (in accordance with Bayliss et al. (2009), Bayliss and Tipper (2006), and Rogers et al. (2014)).

## RESULTS

### Gaze-cueing reaction times

**Figure 2.**
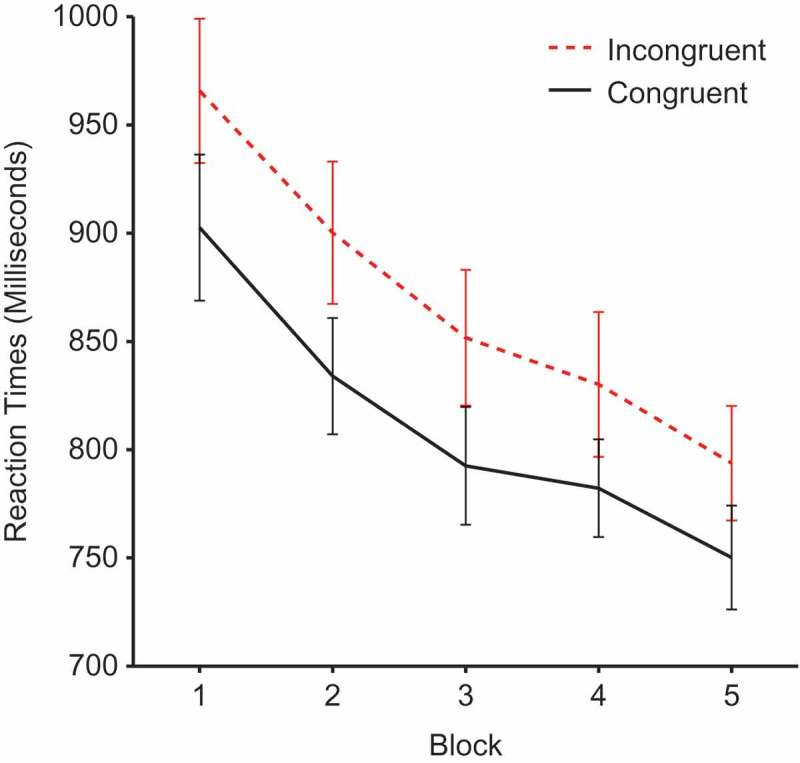
Mean reaction times by block and validity. Error bars show ±1 standard error of the mean.

Reaction times during the cueing phase were submitted to a 2 × 2 × 5 repeated-measures analysis of variance (ANOVA) with factors of face gender, validity and block. There was a significant main effect of validity, *F*(1, 25) = 21.38, *p* <.0001, *η*_p_^2^ = .461, demonstrating a cueing effect due to slower reaction times for incongruent (*M* = 868, *SEM* = 29.74) compared to congruent faces  (*M*= 812, *SEM* = 24.51) (see Figure 2). There was also a significant main effect of block, *F*(1, 25) = 34.089, *p* < .0001, *η*_p_^2^ = .577, and a significant linear trend for block, *F*(1, 24) = 53.04, *p* < 0001, *η*_p_^2^ = .681, demonstrating a general decrease in reaction times as participants become more practiced. However, there was no validity × block interaction, *F*(4, 100) ;= .584, *p* = .675, *η*_p_^2^ = .023, showing that the effects of validity remained constant throughout the experiment. No other effects reached significance.

### Evaluations of trustworthiness

**Figure 3.**
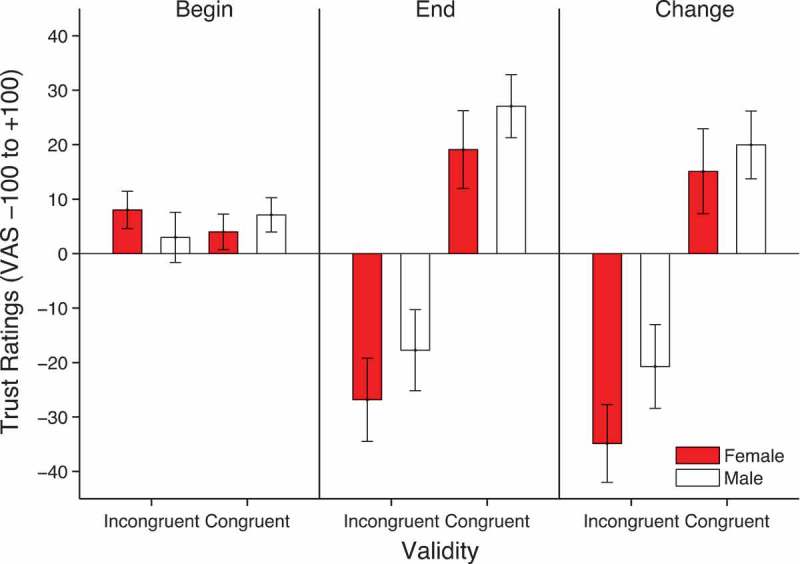
Mean trustworthiness ratings at the beginning and end as well as the change in ratings from beginning to end (end-beginning). Error bars show ±1 standard error of the mean.

The ratings were analyzed using a 2 × 2 × 2 repeated-measures ANOVA with factors of time of rating, face gender and validity. There was a significant main effect of validity, *F*(1, 25) = 15.021, *p* = .001, *η*_p_^2^ = .375, qualified by a significant time × validity interaction, *F*(1, 25) = 16.749, *p *< .0001, *η*_p_^2^ = .401. This is due to more negative ratings for incongruent faces (*M* = −22.29, *SD* = 7.03) compared to congruent faces (*M* = 23.09, *SD* = 5.88) in the final rating phase (see Figure 3). There was also a significant time × face gender interaction, *F*(1, 25) = 4.256, *p *= .050, *η*_p_^2^ = .145. This interaction is due to an overall more negative change in ratings for female (*M* = −9.9, *SD* = 4.4) compared to male faces (*M* = −.38, *SD* = 3.98). No other effects reached significance. In order to formally identify the source of the main effects and interactions described above, separate validity × face gender ANOVAs were run on the beginning and end ratings. These analyses showed that, whereas at the beginning there was no significant effects of validity, *F*(1, 25) = .000, *p *= .985, *η*_p_^2^ = .000, face gender, *F*(1, 25) = .049, *p* = .826, *η*_p_^2^ = .002, or their interaction, *F*(1, 25) = 2.6, *p *= .119, *η*_p_^2^ = .094, at the end rating, there was a significant effect of validity, *F*(1,25) = 16.66, *p *< .0001, *η*_p_^2^ = .40, a significant effect of face gender, *F*(1, 25) = 4.39, *p *= .046, *η*_p_^2^ = .149, but no interaction, *F*(1, 25) = .024, *p* = .879, *η*_p_^2^ = .001.

### Passive viewing phase ERPs

**Figure 4.**
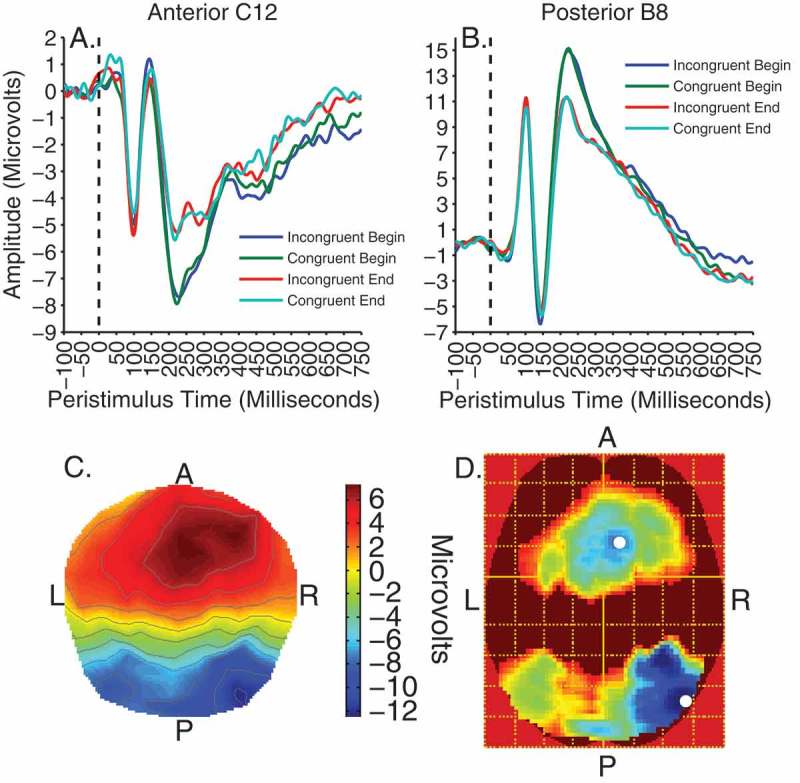
Viewing trials. Top panels illustrate time courses of activity, showing the effect of time at 250 ms on anterior cluster peak electrode C12 **(A)** and posterior peak electrode B8 **(B).** Bottom panels show the scalp distribution of activity for the effect of time at 250 ms **(C**), and the *F*-map of significant voxels **(D)**, thresholded with a voxelwise height threshold of *F* = 15.79, *p* < .0001, uncorrected. Both clusters were clustersize significant at *p *< .0001 RFT FWEC. Colder colors indicate higher *F*-values. White spots denote peak locations.

For analysis of the viewing data, contrasts of interest were time (before/after cueing) and validity × time. To begin with, all effects were tested at an uncorrected threshold of *F*(1, 192) = 6.77, *p* < .01. There were no significant main effects or interactions apart from a strong main effect of time peaking at approximately 250 ms. This contrast was subsequently voxelwise thresholded at *F*(1, 192) = 15.79, *p* < .0001, uncorrected. The effect of time was evident as two significant clusters of activity on separate posterior occipitotemporal and frontocentral electrodes between 200 and 300 ms, peaking at 248 ms, *F*(1, 192) = 40.25, *p* < .0001, *k* = 43,386, for the former and 242 ms for the latter, *F*(1, 192) = 32.58, *p* < .001, *k* = 36,790. Figure 4 shows these clusters of significant activity and illustrates the waveforms for the electrodes nearest to peak voxels in the anterior (electrode C12) and posterior clusters (electrode B8). The effect is characterized by a larger negative deflection at the end compared to the beginning on posterior sites and a larger positive deflection to end compared to beginning on anterior sites. The difference between clusters reflects the typical dipolar distribution of the source of the activity and conforms to the previously reported N250 component (Joyce & Rossion, 2005; Pierce et al., 2011; Schweinberger, Huddy, & Burton, 2004; Schweinberger et al., 2002; Tanaka et al., 2006). The graphs also show the P1, N170 and P200 visual evoked components on posterior electrodes and the N1, VPP and N200 inverted counterparts on frontocentral electrodes. However, no significant effects within the time-windows of the P1, N1, N170 and VPP were observed even at low uncorrected thresholds of *p* < .05.

### 3D source reconstruction

**Figure 5.**
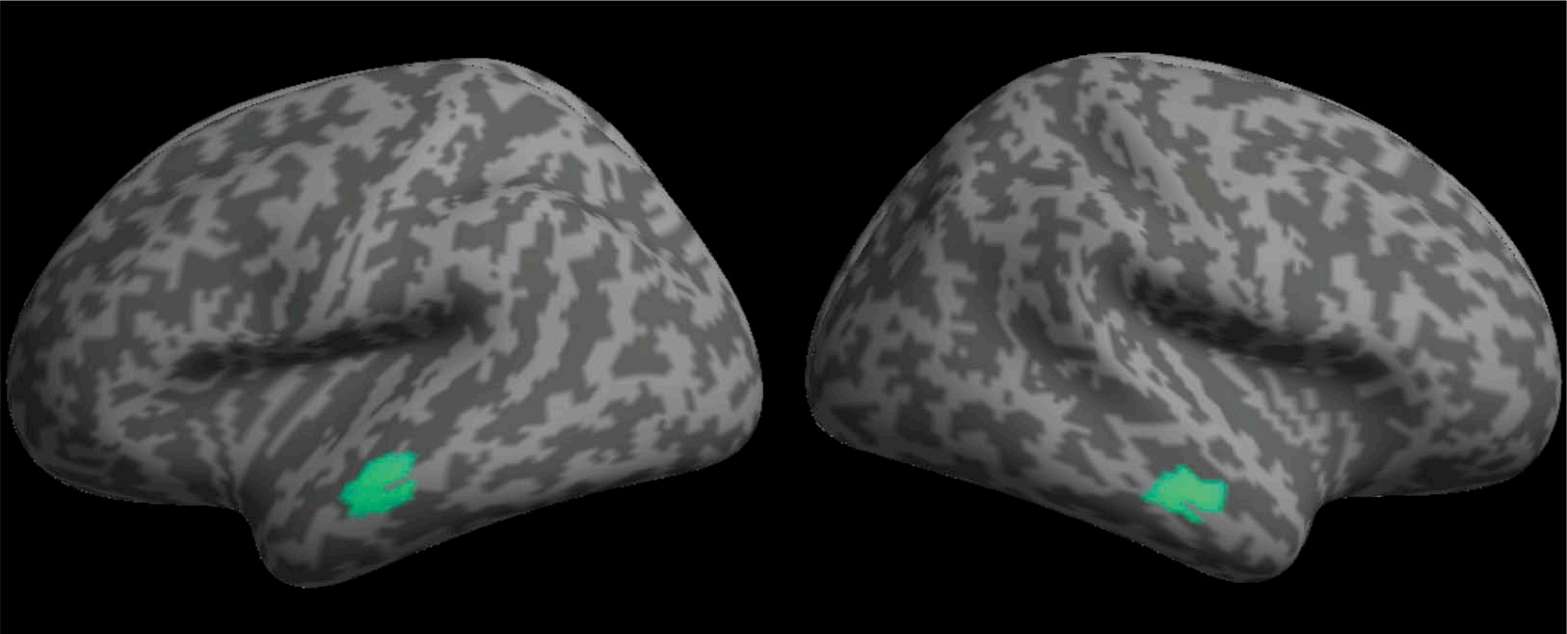
MSP localization of the main effect of time at 250 ms rendered onto a flattened image of the cortical surface (voxelwise thresholded at *p* < .001 RFT FWEC).

Multiple sparse priors were used to model the source of the effect of time at 250 ms during the passive viewing phases. No smoothing was used to preserve the spatial specificity of effects to the cortical surface. This revealed two highly significant bilateral clusters of activity on the anterior middle temporal gyrus (see Figure 5). The effect appeared to be almost symmetrical across hemispheres in both location and magnitude (see Table 1). One peak in each cluster was identified, suggesting all voxels had equal strength.

**TABLE 1:** MNI coordinates, size, and significance of sources of the time effect at 250 ms (*p* < .001, RFT FWEC)

Location	Number of voxels	Peak significance (FWEC)	MNI coordinates of peak		
			X	Y	Z
R middle temporal gyrus	178	*p *< .0001	−64	−18	−10
L middle temporal gyrus	173	*p *< .0001	64	−20	−16

### Gaze-cueing phase ERPs

**Figure 6.**
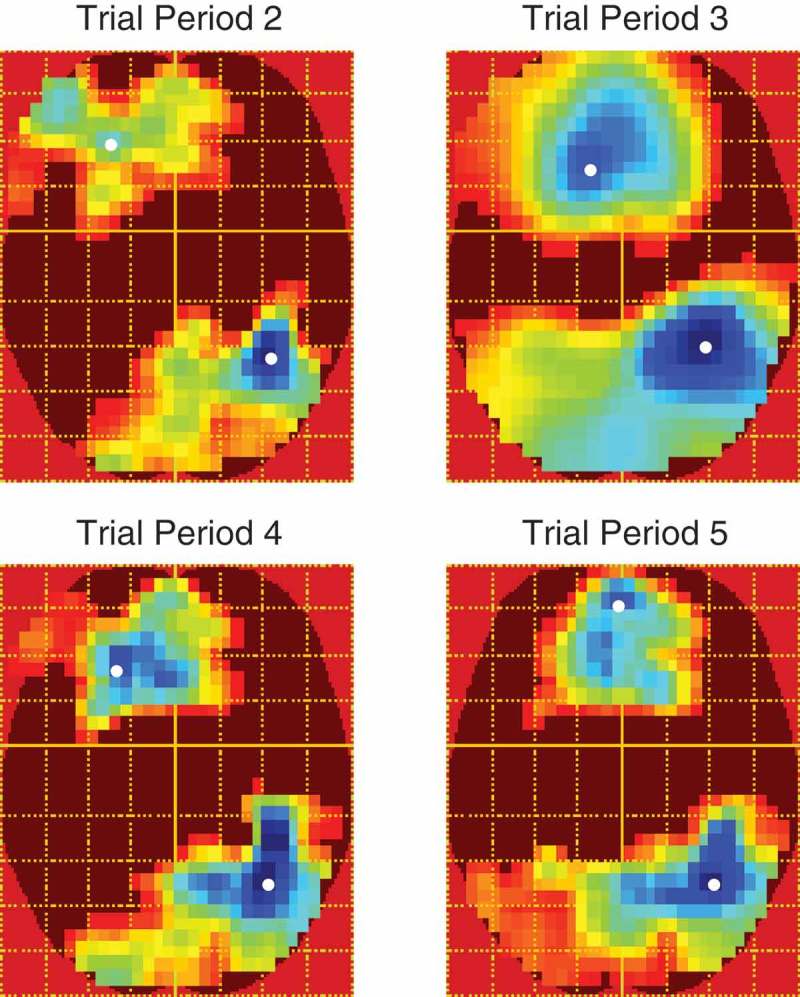
*F*-maps for the linear block × validity interaction across trial periods. All maps are voxelwise thresholded at *F*(1, 480) = 6.69, *p* < .01, uncorrected. However, all clusters were significant at *p* < .0001 RFT FWEC. Colder colors indicate higher *F*-values. White spots show peak voxels.

**Figure 7.**
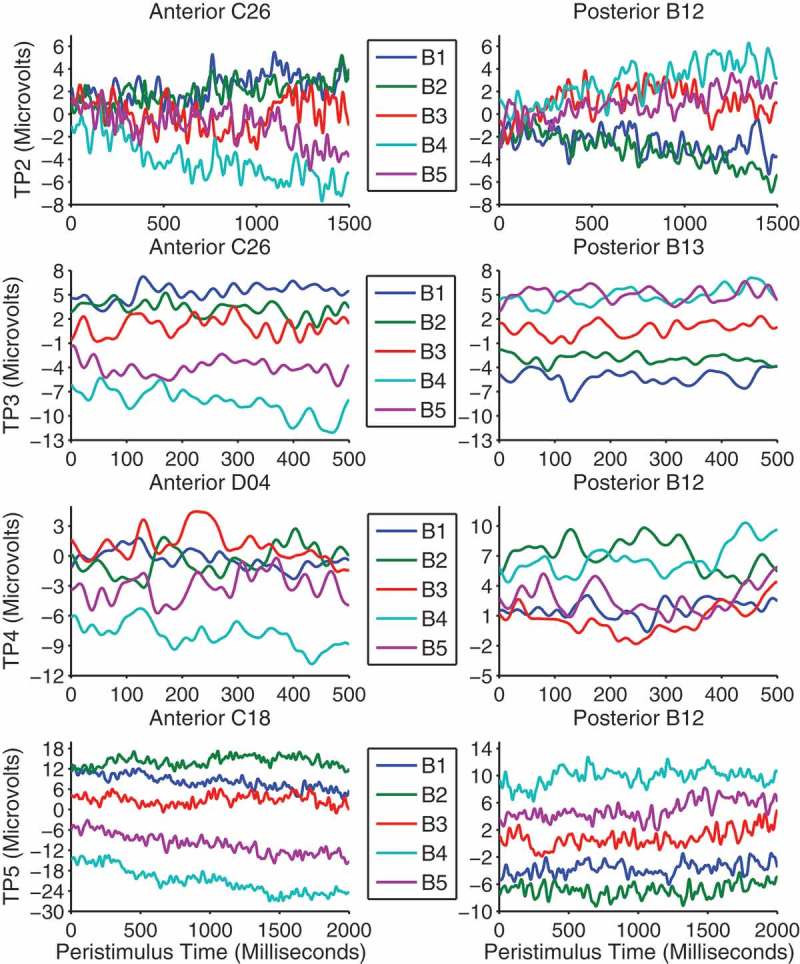
Difference waves between validity conditions (incongruent–congruent) across blocks are shown separately for each trial period on the peak anterior cluster (left panels) and posterior cluster (right panels) electrodes.

In the gaze-cueing phase, each trial period was analyzed separately and all contrasts were voxelwise thresholded at *F*(1, 480) = 6.69, *p *< .01, uncorrected. Contrasts of interest were those pertaining to validity and block × validity. We investigated block × validity with linear contrast weights such that from blocks 1 to 5 congruent faces were weighted as 2, 1, 0, −1 and −2, whereas the sign of the weights were reversed for incongruent faces. Although there were no main effects of validity, there was a significant linear block × validity interaction which emerged in the final 500 ms of trial period 2 and remained almost constant throughout the other trial periods. The distribution of activity across the scalp was similar across trial periods and was evident as two large clusters of activity on separate frontal and parieto-occipital electrodes which were opposite in polarity resembling a typical dipolar pattern (see Figure 6). Figure 7 shows the difference waves (incongruent–congruent) between validity conditions across blocks and trial periods on the electrodes nearest peak voxels in both clusters. The LPP is typically measured as an enhanced positivity over parietal sites (Schupp et al., 2000). Therefore, the patterns of responses are consistent with the interpretation of a gradual increase in the LPP to incongruent faces across blocks despite the polarity of the effect being reversed on anterior electrodes. On posterior electrodes in trial periods 2, 3 and 5, the effect appears to be due to larger LPPs to congruent faces in the first two blocks where after the LPP flips and increases for incongruent faces in blocks 3, 4 and 5. Trial period 4 shows a slightly different pattern and appears to be due to block 3 being larger than blocks 1 and 2 and block 5 being larger than blocks 2 and 4. The differences between trial periods 2, 3, and 5 and trial period 4 is likely due to the extra trials removed, varying trial lengths and differing processes involved in the latter. Also, this is the time point at which a lateral eye movement is made when categorizing the object and thus is more susceptible to distortion by these eye movements and by their removal with ICA. We note that the final ~100 ms of trial period 4 was not significant in the posterior cluster. Table 2 shows the peak time, *F*-values and extent of the effects in voxel size across trial periods and clusters.

**TABLE 2:** Peak times, *F*-values and size of significant clusters across trial periods

	Anterior cluster			Posterior cluster		
Trial period	Peak (ms)	Peak F-value	Size (Voxels)	Peak (ms)	Peak F-value	Size (voxels)
TP2	1454	14.11	41,240	1160	18.82	40,182
TP3	132	24.86	101,354	443	30.88	115,544
TP4	38	15.19	45,662	225	17.70	48,962
TP5	687	16.8	171,374	1485	20.37	279,238

## DISCUSSION

In this experiment we used EEG to examine electrophysiological correlates of emotion during learning of trust from identity-contingent gaze-cues. In addition to standard gaze-cueing effects (Driver et al., 1999; Friesen & Kingstone, 1998; Frischen et al., 2007), we replicated the effects of these cues on trust judgments as incongruent faces were judged less trustworthy than congruent faces (Bayliss et al., 2009; Bayliss & Tipper, 2006; Rogers et al., 2014). During gaze-cueing, we also found that the LPP, related to emotion processing, increased to incongruent faces across blocks after an initial response to congruent faces in the early blocks. This effect is highly consistent with previous research showing that the LPP is modulated by both positive and negatively valenced stimuli (Cuthbert et al., 2000; Schupp et al., 2000) and is related to emotional learning (Franken, Huijding, Nijs & Van Strien, 2011; Sánchez-Nàcher, Campos-Bueno, Sitges, & Montoya, 2011) and memory (Smith, Dolan, & Rugg, 2004).

During gaze-cueing it is important to note that trial periods 2 and 3 are qualitatively different from periods 4 and 5. In the former trial periods 2 and 3 any differences between congruent and incongruent faces must be because of prior learning of the association between face identity and gaze congruency. This is because, in trial period 2, when the face is initially looking straight ahead, and in trial period 3, when gaze has shifted, the absence of a target means participants cannot tell whether this will be a congruent or incongruent trial. Only via retrieval of prior episodes of gaze-cueing behavior evoked by the particular viewed face, can the validity of the face be known in advance of the target. It would therefore appear that retrieval of a face’s prior gaze-cueing behavior takes approximately 1000 ms, as it is only in the last 500 ms of trial period 2 that significant LPP effects are detected. In contrast, trial period 4 reflects the period where gaze is directed toward or away from the target, so there is an explicit signal as to whether the face deceives or not; while trial period 5 reflects the situation where review of the previous congruent or incongruent face can take place.

The significant change in EEG activity between blocks 2 and 3, as the experiment progresses would appear to be the time when a qualitative change in the representation of the faces takes place. That is, the time when the salience of the incongruently gazing face that is misleading and deceiving the participant is represented. However, the effect appeared not to be perfectly linear. In all trial periods the largest LPP difference to incongruent was observed in block 4 and in trial period 5, after the gaze-cue occurred, the largest response to congruent faces occurred in block 2. This suggests a role for learning and habituation. Thus, learning about the trustworthiness of congruent faces may peak in block 2 before habituating where after learning about the trustworthiness of incongruent faces proceeds until block 4 when the response to incongruent faces also begins to habituate. Such habituation during the final block may explain why no effects of validity were observed in the final passive viewing phase, despite a large N250 face familiarity effect.

The peak of both the cueing LPP and viewing N250 effects appeared to localize to the right posterior hemisphere. This is highly consistent with both the emotion and face processing literature. The core face processing areas of the occipital and temporal lobes (the occipital face area, fusiform face area and superior temporal sulcus) are right hemisphere dominant (Kanwisher & Barton, 2011) and the right hemisphere has been proposed to be specialized for emotion processing (Silberman & Weingartner, 1986) or processing negative emotions (Harmon-Jones, Gable, & Peterson, 2010). For both reasons, the LPP effects related to faces may be more right hemisphere distributed, when displayed with an average reference. The LPP has been proposed to be due to enhanced attention to motivating stimuli (Hajcak et al., 2010) and this may be reflected in increased processing in category specific brain regions, shifting the scalp distribution of peak activity to the right hemisphere.

The increase in the LPP to incongruent faces across blocks is in line with the notion that participants initially anticipate a pro-social, trustworthy, interaction, as shown in the initial explicit trust ratings, but that learning has to gradually occur as expectancies are repeatedly violated. Previous studies have shown that the LPP to oddball negative stimuli is larger than to oddball positive stimuli among more frequently presented neutral stimuli during evaluation (Hilgard, Weinberg, Proudfit, & Bartholow, 2014; Ito, Larsen, Smith, & Cacioppo, 1998). In addition, untrustworthy faces elicit a larger LPP than trustworthy faces (Marzi et al., 2014; Yang et al., 2011). Our findings also suggest that negative, untrustworthy, incongruent cues are given greater weight than the positive, trustworthy, congruent cues. We repeated our analysis using the linear contrast weights separately for the block effect for incongruent and congruent faces. All clusters in all trial periods were significant for incongruent but not congruent faces. The LPP is reduced when attention is cued to a non-arousing portion of unpleasant pictures (Dunning & Hajcak, 2009; Hajcak, Dunning, & Foti, 2009). Therefore, the linear effect may be due to increased attentional salience by emotion to the negatively judged incongruent faces as blocks progress. In turn, this may facilitate the learning of face valence to produce changes in trust judgments and may explain the increased feelings of familiarity for incongruent compared to congruent faces (Bayliss & Tipper, 2006). The LPP in response to unpleasant images is also reduced after cognitive reappraisal, where the image is reinterpreted in a less negative way (Foti & Hajcak, 2008; Hajcak & Nieuwenhuis, 2006). The interaction of the LPP with cognition may relate the LPP to appraisal processes at rating.

We did not assess participant’s conscious awareness of the contingencies between face identity and gaze-cues. However, this issue has been addressed by similar studies (Bayliss et al., 2009; Rogers et al., 2014) where pairs of matched faces were presented to participants after gaze-cueing. One of the faces had always gazed congruently whereas the other had always gazed incongruently. When participants judged which of the two faces was more likely to look toward the target, they chose incongruent faces equally as frequently as congruent faces. Here, we also found the gaze-cueing effect was still evident in block 5 after repeated exposures to the identity-contingent gaze-cues. This suggests that participants were not using the identity and gaze direction of the face to anticipate target location, which would be expected if participants had conscious knowledge of the contingencies between identity and gaze-cues. However, we do not make strong claims concerning awareness of gaze contingencies, as there were some changes to the procedure, such as the measurement of initial ratings of trust, and we did not explicitly investigate the awareness issue.

In the passive viewing trials, faces were presented at the start of the experiment to provide a baseline measure of face-related ERPs, and then at the end of the experiment the faces were again passively viewed in an attempt to detect whether the faces that had consistently looked at targets, congruently, could be discriminated from those consistently looking away, incongruently. The results from the passive viewing conditions confirmed previous findings concerning face repetition/familiarity. We found a strong N250 familiarity effect, where the ERP signal around 250 ms on posterior occipitotemporal sensors was significantly changed from first viewing of faces relative to viewing at the end of the experiment after numerous exposures. Interestingly, exploratory source localization using MSPs clearly identified bilateral anterior temporal (ATL) cortical sources for the N250. This is in contrast to earlier studies suggesting a more posterior fusiform gyrus source (Schweinberger, Kaufmann, Moratti, Keil, & Burton, 2007; Schweinberger et al., 2002) using the brain electrical source analysis (BESA) approach. However, our findings are highly consistent with fMRI and single-unit recording studies in both macaque monkeys and humans (Freiwald & Tsao, 2010; Tsao, Moeller, & Freiwald, 2008). For example, in humans, multivoxel pattern classification of fMRI data has found voxels in ATL that can reliably differentiate between different faces (Kriegeskorte, Formisano, Sorger, & Goebel, 2007) and activity in the same region of ATL correlates with behavioral measures of face recognition performance (Nasr & Tootell, 2012).

However, our main concern was to identify the neural signal for face-trust learning. We know from participants’ explicit reports that the gaze-cueing procedure significantly changed their trust ratings of the faces. Yet in the analysis of the ERP response during passive viewing of the faces we found no evidence for such discrimination. We believe that the contrast between behavior and neural activity is because the faces in the passive viewing procedure were presented for a relatively brief period of 750 ms. The analysis of the gaze-cueing procedure suggests that the statistically significant discrimination of different trust assessments emerges after 1000 ms. Furthermore, as noted above, there may have been habituation processes which could have reduced detection of gaze-cue validity toward the end of the experiment when participants passively viewed the faces with no explicit task. Alternatively, the N250 and LPP in the end-viewing phase may have been equally sensitive to the affective qualities of both congruent and incongruent faces, but the reason no difference was observed during passive viewing may be due to a bivalent response profile.

In conclusion, here we presented unique data from EEG as evidence for the role of emotion in the learning of trustworthiness from gaze-cues. We found that the emotion-related LPP increased across blocks for incongruent compared to congruent faces possibly reflecting increased emotion, attention to and learning about, faces that deceive. The neural signature for this encoding of deceptive incongruent gaze-cueing behavior appeared to emerge between blocks 2 and 3. The discrimination of congruent and incongruent faces in early periods of the trial reflects retrieval of prior gaze-cueing behavior and this retrieval process takes approximately 1000 ms to emerge.

No potential conflict of interest was reported by the authors.

## APPENDIX

Using repeated-measures ANOVA on the mean number of repetitions of each face in the viewing phase, there was no effect of validity (*F*(1, 25) = .151, *p* = .701, *η*_p_^2^ = .006), time (*F*(1, 25) = 0.0, *p* = 0.0, *η*_p_^2^ = 0.0) or there interaction (*F*(1, 25) = 1.08, *p *= .309, *η*_p_^2^ = .041). Thus, the effects would be similar to that of removing trials due to artifacts.[Table T0003] [Table T0004]

**TABLE A1 T0003:** Mean number of repetitions of identities and trials in each condition in the analyses of the viewing phases

	Beginning		End	
	Congruent	Incongruent	Congruent	Incongruent
Repetitions mean	5.9	6.15	6.03	5.97
Repetitions SD	.535	.535	.54	.54
Number of trials mean	42.58	44.23	42.89	41.92
Number of trials SD	6.01	6.27	7.39	6.25

**TABLE A2 T0004:** Mean numbers of trials available in the cueing analysis

	Block 1	Block 2	Block 3	Block 4	Block 5
Congruent	11.77(2.07)	11.39(3.37)	11.70(3.33)	12.08(2.33)	11.96(3.07)
Incongruent	11.65(3.51)	11.77(2.94)	11.54(2.98)	11.15(2.87)	11.69(2.94)
